# Voltage-gated Na^+^ Channel Activity Increases Colon Cancer Transcriptional Activity and Invasion Via Persistent MAPK Signaling

**DOI:** 10.1038/srep11541

**Published:** 2015-06-22

**Authors:** Carrie D. House, Bi-Dar Wang, Kristin Ceniccola, Russell Williams, May Simaan, Jacqueline Olender, Vyomesh Patel, Daniel T. Baptista-Hon, Christina M. Annunziata, J. Silvio Gutkind, Tim G. Hales, Norman H. Lee

**Affiliations:** 1 Department of Pharmacology and Physiology, The George Washington University, Washington, DC; 2 The National Institute of Dental and Craniofacial Research, Bethesda, MD; 3 The Institute of Academic Anaesthesia, Division of Neuroscience, Ninewells Hospital, University of Dundee, Dundee, DD1 9SY, UK; 4 Women’s Malignancies Branch, National Cancer Institute, Bethesda, MD.

## Abstract

Functional expression of voltage-gated Na^+^ channels (VGSCs) has been demonstrated in multiple cancer cell types where channel activity induces invasive activity. The signaling mechanisms by which VGSCs promote oncogenesis remain poorly understood. We explored the signal transduction process critical to VGSC-mediated invasion on the basis of reports linking channel activity to gene expression changes in excitable cells. Coincidentally, many genes transcriptionally regulated by the *SCN5A* isoform in colon cancer have an over-representation of *cis*-acting sites for transcription factors phosphorylated by ERK1/2 MAPK. We hypothesized that VGSC activity promotes MAPK activation to induce transcriptional changes in invasion-related genes. Using pharmacological inhibitors/activators and siRNA-mediated gene knockdowns, we correlated channel activity with Rap1-dependent persistent MAPK activation in the SW620 human colon cancer cell line. We further demonstrated that VGSC activity induces downstream changes in invasion-related gene expression via a PKA/ERK/c-JUN/ELK-1/ETS-1 transcriptional pathway. This is the first study illustrating a molecular mechanism linking functional activity of VGSCs to transcriptional activation of invasion-related genes.

Membrane-spanning voltage-gated Na^+^ channels (VGSCs), important for neurite extension, neurotransmitter release and cardiac excitation-contraction coupling, have been implicated in the metastatic potential of several different cancers. VGSCs are responsible for the depolarization phase of the action potential in excitable cells, such as neurons and cardiomyocytes, where their activation leads to an influx of Na^+^ into the cell^1^. Aberrant Na^+^ homeostasis can affect intracellular Ca^2+^ concentrations and pH, conditions that can alter intracellular signaling pathways. VGSC activity participates in the oncogenic potential of lung, prostate, breast, and colon cancers^2^,^3^,^4^,^5^,^6^,^7^,^8^. Importantly, these studies demonstrate that channel activity leads to increased invasion, whereas pharmacological blockade or genetic knockdown of specific VGSC isoforms inhibits invasion. The mechanism(s) by which these channels confer an oncogenic advantage remains unclear. Fluxes in pH can lead to extracellular acidification thereby enhancing the proteolytic activity of pH-dependent cathepsins for cellular invasion. It has been shown that the Na^+^/H^+^ exchanger type 1 partners with Na_V_1.5 to regulate H^+^ efflux and enhance invasiveness in breast cancer cells^9^,^10^. A recent study showed that Na_V_1.5 allosterically regulates NHE-1 to promote invadopodia formation and activity in breast cancer cells^11^. A study investigating melanoma cell invasion demonstrated that activation of the Na_V_1.6 VGSC increased intracellular Ca^2+^ release and invadopodia formation^12^. It has also been proposed that membrane depolarization supports cancer stem cell populations by preventing maturation and/or differentiation^13^.

We have previously demonstrated that expression of the Na_V_1.5 isoform (encoded by *SCN5A*) is restricted to colon cancer tissues when compared to normal-matched controls and functional activity contributed to invasiveness of colon cancer cell lines^4^. Our work further showed that *SCN5A* participates in a colon cancer invasion-related gene network and regulates the expression of genes important for invasion. Lacking from these studies has been a comprehensive investigation of the signal transduction pathway(s) facilitating the oncogenic phenotype following VGSC activation.

The evolutionarily conserved mitogen activated protein kinase (MAPK) pathway, deregulated in many cancers, integrates extracellular signaling to cellular responses, such as replication, differentiation and apoptosis^14^. The MAPK signaling pathway is commonly activated in colon cancers and it has been suggested that all kinases (e.g. RAF, MEK and ERK isoforms) participating in this pathway could be potential targets for therapy^15^. In neurons, initiation of membrane depolarization by voltage-gated ion channels leads to activation of the ERK1 and ERK2 (ERK1/2) MAPKs, a process important for synaptic plasticity and neurogenesis^16^,^17^,^18^. The upstream signaling molecules responsible for ERK1/2 activation following membrane depolarization can include Ca^2+^ influx, calmodulin, cAMP-dependent protein kinase A (PKA), Ras and Rap1 (small GTPases of the Ras subfamily) and the Raf kinases^16^,^17^,^19^. Once activated, ERK1/2 translocates to the nucleus to phosphorylate and activate transcription factors (TFs), leading to changes in gene expression^20^.

Although evidence suggests that dysregulation of the MAPK pathway is important for colon cancer progression, no studies have examined the possible link between VGSC activity and aberrant stimulation of ERK1/2 in colon cancer cells. If such a link exists, it would also be informative to identify some of the effectors mediating VGSC stimulation of ERK1/2. As cancer cells are known to usurp and express various genes associated with excitable cells, which appear to facilitate invasion^4^,^21^, it is plausible that colon cancer cells may mimic many of the same signal transduction events found in neurons that lead to gene expression changes following ion channel activation. We show here that enhanced VGSC activity in colon cancer cells leads to increased ERK1/2 MAPK activity, whereas blockade of VGSCs had the opposite effect. We further demonstrate that VGSC-mediated stimulation of ERK1/2 was persistent and dependent on PKA, Rap1B (but not Rap1A) and MEK. Moreover, persistent ERK1/2 activation in colon cancer cells was associated with the following: i) enhanced TF binding to the proximal promoter region of genes associated with invasion, ii) transcriptional up-regulation of invasion-related genes, and iii) corresponding increase in invasive activity. Thus, we propose that Na_V_1.5 VGSC activity confers an oncogenic advantage to colon cancer cells by stimulating PKA/Rap1B/MEK/ERK1/2 to transcriptionally up-regulate genes required for invasion.

## Results

**Figure 1:**
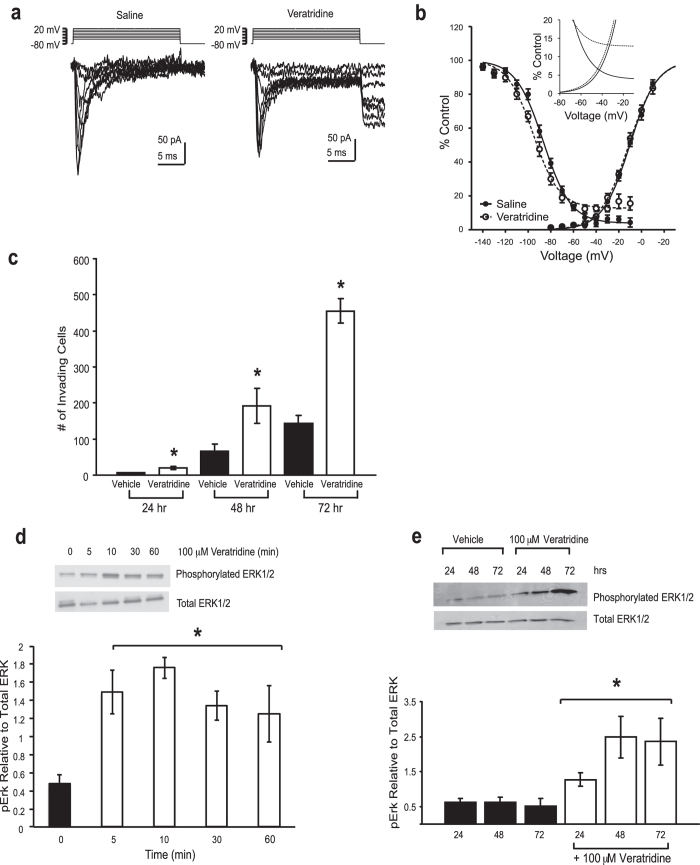
Veratridine-enhanced Na^+^ current, invasion potential, and MAPK signaling. **a)** Representative voltage-activated Na^+^ currents recorded from voltage-clamped SW620 cells in the absence and presence of 100 μM veratridine. Currents were activated by depolarizing to voltages between −70 and +20 mV in 10 mV steps. **b)** A graph depicting the voltage-dependence of activation and inactivation of conductance mediated by VGSCs in the absence (closed circles) or presence (open circles) of 100 μM veratridine expressed as a percentage of maximal conductance. Data were fitted with a Boltzmann function, as described previously^22^. Values for V½ of activation and inactivation in the absence (−4.9 ± 2.7 and −86 ± 1.5 mV; n = 15, respectively) and presence of veratridine (−2.4 ± 5.3 and −95 ± 3.0 mV; n = 10, respectively) were determined as described in the Methods. The values for V½ of VGSC inactivation in the absence and presence of veratridine differed significantly (p < 0.05, unpaired t-test). The inset graph illustrates the predicted window of available current within the fits to activation and inactivation curves, in the absence (solid lines) and presence (dashed lines) of veratridine. Veratridine increases the size of the window current by reducing the extent of inactivation. **c)** Total number of invading SW620 cells as assessed by Matrigel assay over 24, 48 and 72 hr was significantly increased in the presence of 100 μM veratridine. Vehicle is < 0.1% DMSO final concentration. **d)** Western blot analysis demonstrates increase in activated ERK that occurs with short exposure to veratridine. Note that slight diminution of total ERK1/2 at 5 min is loading artifact. **e)** Western blot analysis demonstrates increase in activated ERK that occurs with prolonged exposure to veratridine. Traces are representative of 3 independent experiments. Columns, mean from at least 3 independent experiments; bars, SEM. * significantly different compared with vehicle control, P < 0.05, two-sided unpaired t-test (c); * significantly different compared with vehicle control (0 min), P < 0.05, ANOVA with post-hoc Dunnett’s (d); * significantly different compared to 24, 48 and 72 hr vehicle control, P < 0.05, ANOVA with post-hoc Holm test, all other pairwise comparisons non-significant (e).

Veratridine binds to activated VGSCs and prevents their inactivation leading to aberrant Na^+^ influx^21^. The effect of veratridine on VGSCs expressed in colon cancer cells is unknown. Therefore, we used the whole-cell recording technique to examine Na^+^ currents in voltage-clamped SW620 cells. We have previously demonstrated that depolarization activates VGSCs in SW620 cells with a maximum conductance activated at 20 mV^4^. Here we determined whether veratridine modulates Na^+^ currents recorded from SW620 cells. Consistent with our previous work, depolarization activates robust VGSC currents in SW620 cells (Fig. 1a). Application of veratridine (100 μM) led to an enhancement of the steady-state current, as well as the appearance of a tail current upon repolarization (Fig. 1a). The net effect was a substantial increase in persistent Na^+^ current. We generated activation curves by plotting the conductance (normalized to maximum) at each voltage, in the absence or presence of veratridine (Fig. 1b). A Boltzmann function was fitted to the data to determine V½ values. Veratridine did not alter the V½ of activation in VGSCs expressed in SW620 cells. To examine the effects of veratridine on steady-state inactivation, SW620 cells were exposed to 100 ms pre-pulses to between −140 mV and −10 mV. The amount of available current was determined by stepping to 0 mV. Current values were converted into conductance and plotted as a percentage of maximum (Fig. 1b). The fit of the Boltzmann function to the steady-state inactivation data reveals that veratridine caused a small hyperpolarizing shift in the V½ of inactivation. This was accompanied by a reduction in the extent of inactivation increasing the amount of available current at more depolarized potentials ( > −45 mV). The amount of current that was resistant to inactivation (as a percentage of peak current) was significantly increased in the presence of veratridine (12 ± 2.2%; n = 10), when compared with controls (5.1 ± 1.9; n = 15; p < 0.05; unpaired t-test). The superimposed activation curves reveal the window current (Fig. 1b **inset**), which was increased by vetratridine.

We next determined whether veratridine could increase the invasive potential of colon cancer cells. In keeping with our previous study the ability of SW620 cells to invade Matrigel was significantly enhanced by veratridine^22^. Veratridine (100 μM) enhanced invasion over a 24, 48 and 72 hr time period compared to vehicle control (Fig. 1c). Correspondingly, the veratridine-stimulated invasion was associated with a persistent activation of the MAPKs (beginning at 5 min and lasting up to 72 hr), as shown by western blot analysis with an antibody specific to the phosphorylated (i.e. activated) form of ERK1/2 (Fig. 1d,e). This observation of MAPK pathway stimulation in response to VGSC activity in colon cancer cells is analogous to previous findings in neurons and more recently in endothelial cells^16^,^23^.

**Figure 2:**
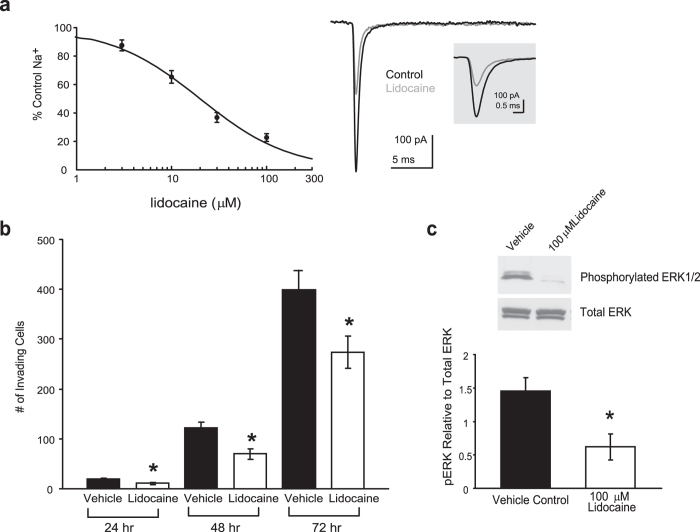
Inhibition of VGSC current reduces invasion potential and MAPK signaling. **a)** Lidocaine concentration-response relationship for inhibition of Na^+^ currents recorded from SW620 colon cancer cells. Currents were activated by stepping from a holding potential of −80 mV to 10 mV. The inhibition was measured at the peak of the current. Data, fitted by using the logistic equation to determine the IC_50_ value for lidocaine, are presented as mean ± SEM of 5 independent experiments for each data point. The exemplar current reveals the inhibitory effect of 10 μM lidocaine. The current is expanded to illustrate the inhibition of the peak current. **b)** Total number of invading SW620 cells over 24, 48 and 72 hr as assessed by Matrigel assay was significantly decreased in the presence of 100 μM lidocaine, a local anesthetic that blocks VGSCs. Vehicle is <0.1% ethanol final concentration. **c)** Western blot analysis demonstrates the loss of activated ERK that occurs with 72 hr lidocaine (100 μM) treatment. A representative western blot is shown (top). Results are means ± SEM from at least 5 independent experiments (a, b and c). * Significantly different compared with vehicle control, P < 0.05, two-sided unpaired t-test.

Having established that veratridine stimulates Na^+^ currents, invasion and persistent activation of ERK1/2 in colon cancer cells; we next investigated the effects of a VGSC blocker, lidocaine, on the aforementioned oncogenic parameters. We confirmed the ability of lidocaine to dose-dependently inhibit voltage-activated Na^+^ currents in SW620 cells. Na^+^ currents were activated by depolarizing from −80 mV to 10 mV and lidocaine was applied to the recording chamber. The inhibitory effect of lidocaine on the peak Na^+^ current amplitude was established to produce a concentration-response relationship from which the IC_50_ was determined to be 19.9 + 2.5 μM, with 100 μM causing approximately 80% loss of current (Fig. 2a). The total number of invading SW620 cells was significantly reduced in the presence of 100 μM lidocaine compared with vehicle at 24, 48 and 72 hr (Fig. 2b). Lastly, 72 hr treatment of SW620 cells with 100 μM lidocaine led to a significantly lower level of activated ERK1/2 compared to vehicle control (Fig. 2c).

**Figure 3:**
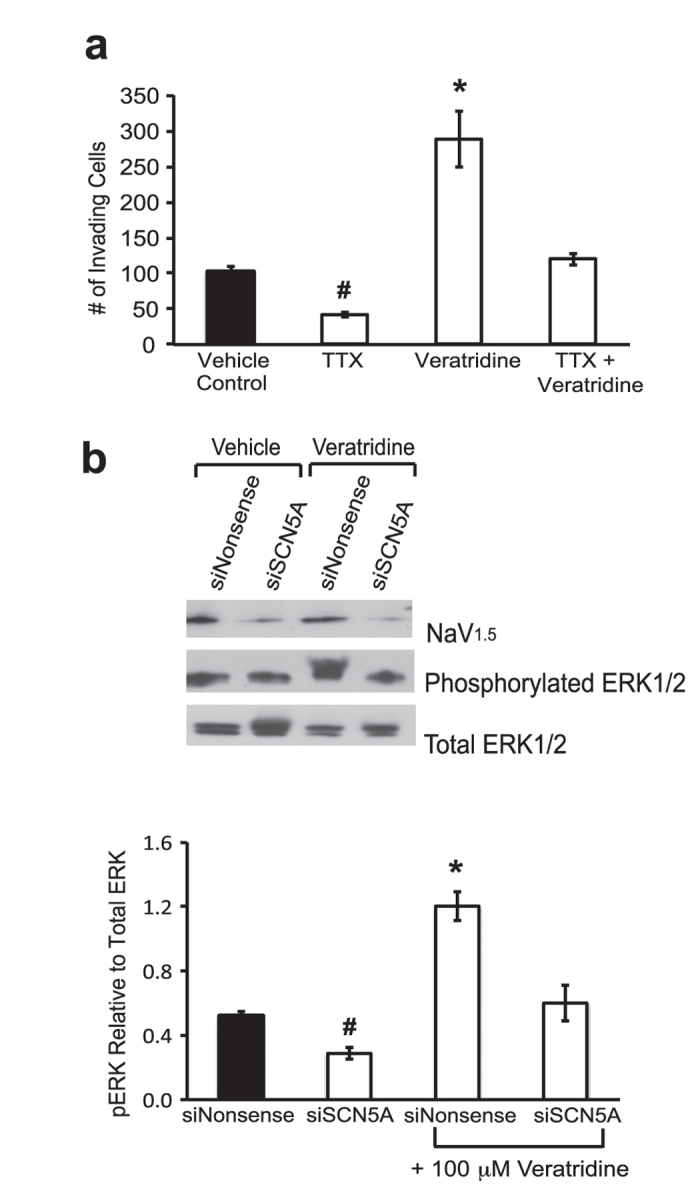
Invasion potential and MAPK signaling is mediated by Na_v_1.5 isoform. **a)** Total number of invading cells is significantly decreased with 30 μM TTX and significantly increased in the presence of 100 μM veratridine over a 48 hr time period. Veratridine-stimulated invasion is abrogated in the presence of TTX. **b)** siRNA-mediated knockdown of *SCN5A* significantly reduced activated ERK1/2 levels compared to cells treated with a nonsense siRNA and abrogated veratridine-stimulated ERK1/2 activation. Two separate siRNAs were combined (siSCN5A) for knockdown experiments (the 2 siRNAs were previously demonstrated to lack off-target effects)^4^. A nonsense siRNA (siNonsense) served as control. * Significantly different for all pairwise comparisons, # significantly different for all pairwise comparisons. P < 0.05, ANOVA with post-hoc Holm test (a and b). Results are means ± SEM from at least 3–5 independent experiments for each data point.

As lidocaine has been shown to inhibit, in addition to VGSCs, other membrane-bound signaling molecules involved in oncogenesis^24^,^25^, the following experiments were employed to more conclusively demonstrate a link between VGSC activity and ERK1/2 phosphorylation and resultant invasive activity in SW620 colon cancer cells. Our previous work has demonstrated that Na_V_1.5 VGSC isoform (encoded by *SCN5A*) is responsible for invasivity of colon cancer cells as ‘knockdown’ of this isoform with either tetrodotoxin (TTX), a specific inhibitor of VGSCs, or siRNA-mediated targeting led to a significant loss of baseline invasion^4^. Treatment of SW620 cells with 30 μM TTX alone resulted in a significant >2-fold decrease while, 100 μM veratridine led to a significant increase in the total number of invading cells over a 48 hr time period (Fig. 3a). TTX reversed veratridine-stimulated invasion with the total number of invading cells returning to baseline levels of vehicle control-treated cells (Fig. 3a). Noteworthy was the finding that this reversal did not reach sub-baseline levels seen with TTX treatment alone (compare significant difference between TTX versus TTX + Veratridine in Fig. 3a). Next, the role of Na_V_1.5 in mediating 48 hr veratridine-stimulated phosphorylation of ERK1/2 in SW620 cells was assessed. Targeted siRNA-mediated knockdown of *SCN5A* led to a significant decrease in ERK1/2 phosphorylation (compare siNonsense control versus siSCN5A, Fig. 3b). In cells treated with a scrambled sequence siRNA (siNonsense), veratridine robustly stimulated ERK1/2 phosphorylation 2–3 fold. Analogous to our invasion findings in Fig. 3a, knockdown of *SCN5A* reversed veratradine-stimulated ERK phosphorylation back to baseline but not sub-baseline levels (compare significant difference between siSCN5A versus siSCN5A + veratridine, Fig. 3b). This implies a minor veratridine-stimulated component that is independent of *SCN5A*.

**Figure 4:**
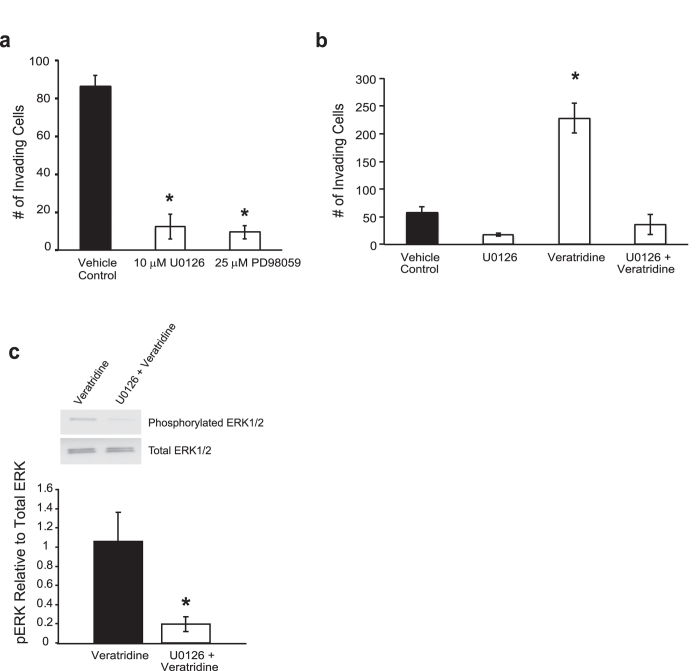
MAPK signaling contributes to invasion potential of colon cancer cells. **a)** Inhibition of MEK by either 10 μM U0126 or 25 μM PD98059 significantly reduces invasion over 48 hr. U0126 treatment abrogates both (**b**) veratridine-stimulated invasion and (**c**) MAPK activity over a 48 hr time period in SW620 cells. Results are means ± SEM from at least 4 independent experiments for each data point. * Significantly different from Vehicle Control, P < 0.05, ANOVA with post-hoc Dunnett’s (a); * significantly different for all pairwise comparisons, P < 0.05, ANOVA with post-hoc Holm test (b); * significantly different from Veratridine, P < 0.05, two-sided unpaired t-test (c).

The findings taken as a whole (Figs 1, 2, 3) suggest that veratridine-stimulated ERK1/2 activity may lead to increased colon cancer cell invasion. To substantiate this hypothesis, ERK1/2 phosphorylation and invasion potential were assessed following inhibition of MEK, the upstream kinase responsible for phosphorylation and activation of ERK1/2^20^. Under baseline conditions (no veratridine stimulation), SW620 cells treated with the MEK inhibitor U0126 at 10 μM or PD98059 at 25 μM led to a significant decrease in invasion over a 48 hr period (Fig. 4a). We then examined whether inhibition of MEK could abrogate veratridine-stimulated invasion and ERK1/2 phosphorylation. In the presence of 10 μM U0126, there was a reversal of veratridine stimulated invasion and a significant decrease in ERK1/2 phosphorylation following 48 hr veratridine stimulation (Fig. 4b,c). These data lend support to the idea that the MAPK pathway is persistently activated in response to VGSC activation in colon cancer cells.

**Figure 5:**
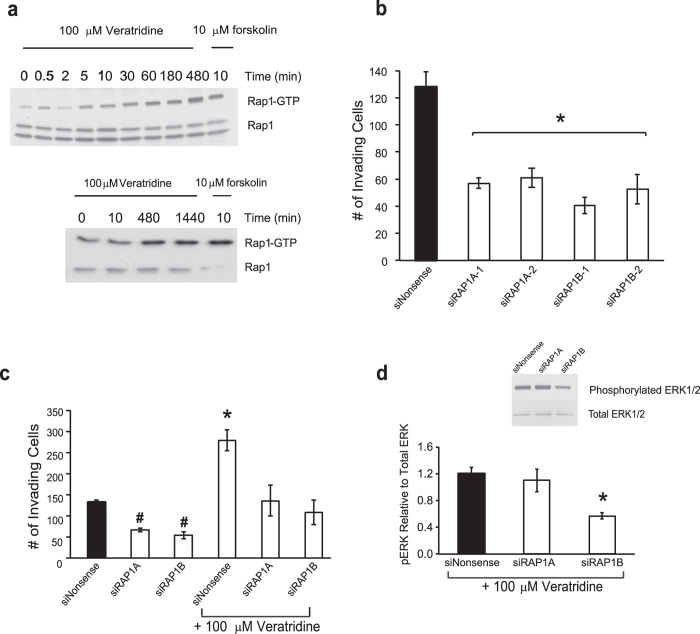
Veratridine signals through Rap1 to enhance invasiveness and persistent MAPK signaling in colon cancer cells. **a)** SW620 cells were serum starved for 24 hr prior to treatment with 100 μM veratridine for time periods indicated. A 10 min, 10 μM forskolin treatment was included as a positive control. Cell lysates were subjected to pulldown assay for detecting activated GTP-bound Rap1. An increase in GTP-bound Rap1 can be seen starting at 5 min and persists through 24 hr. **b)** siRNA-mediated knockdown of *Rap1A* and *Rap1B* inhibited basal SW620 invasion through Matrigel at 48 hr. Two separate siRNAs (−1 and −2) were employed for each Rap1 isotype. A nonsense siRNA (siNonsense) served as control. **c)** siRNA-mediated knockdown of Rap1A and Rap1B inhibited veratridine-stimulated SW620 invasion through Matrigel at 48 hr. **d)** siRNA-mediated knockdown of *Rap1B* (siRAP1B), but not *Rap1A* (siRAP1A), inhibited veratridine-stimulated ERK1/2 activity at 48 hr. Representative western blot provided above bar graph. In the experiments depicted in panels c and d, both siRNAs (−1 and −2) for each respective Rap1 isotype were combined for knockdown experiments, as off-target effects were not observed (see panel b). The combination of siRNAs led to greater knockdown efficiency as defined by qRT-PCR of *Rap1B* and *Rap1B* mRNAs. Results are means ± SEM from at least 4 independent experiments for each data point. * Significantly different from siNonsense control, P < 0.05, ANOVA with post-hoc Dunnett’s (b); * significantly different for all pairwise comparisons, # significantly different for all pairwise comparisons (except for siRAP1A versus siRAP1B), P < 0.05, ANOVA with post-hoc Holm test, all other pairwise comparisons non-significant (c and d).

Rap1, a small G protein important for neurite extension, adhesion and migration, is activated in response to depolarization^16^,^26^,^27^. Moreover, Rap1 activation can lead to persistent stimulation of the MAPK pathway^28^,^29^. To examine the role of Rap1 in the VGSC-mediated signaling in SW620 cells, a pull down assay was used to quantify the level of guanosine 5'-triphosphate (GTP)-bound Rap1 (active form) following 100 μM veratridine treatment. Forskolin, a direct activator of adenylyl cyclase that leads to PKA stimulation and subsequent Rap1 activation, was used as a positive control. There was a significant enhancement in active Rap1 levels as early as 5 min of treatment, and activated Rap1 persisted up to the longest treatment time point assayed, 24 hr (Fig. 5a). Next, SW620 cells were treated with individual siRNAs directed against *Rap1A* and *Rap1B* to determine isoform dependency in basal and 48 hr veratridine-stimulated invasion of SW620 cells. When compared to unstimulated cells treated with a nonsense sequence siRNA (i.e. baseline invasion), unstimulated cells treated with siRNAs for knockdown of either *Rap1A* or *Rap1B* displayed significantly less invasive phenotype over 48 hr (Fig. 5b). Furthermore, knockdown of either *Rap1A* or *Rap1B* led to a reversal of 48 hr veratridine-stimulated invasion back to baseline, but not sub-baseline, levels (Fig. 5c). In parallel with the loss of invasion, there was a significant loss in the levels of active ERK1/2 when 48 hr veratridine was given in the presence of *Rap1B* knockdown (Fig. 5d). In contrast, there was no change in active ERK1/2 levels when 48 hr veratridine was given after knockdown of *Rap1A* (Fig. 5d).

**Figure 6:**
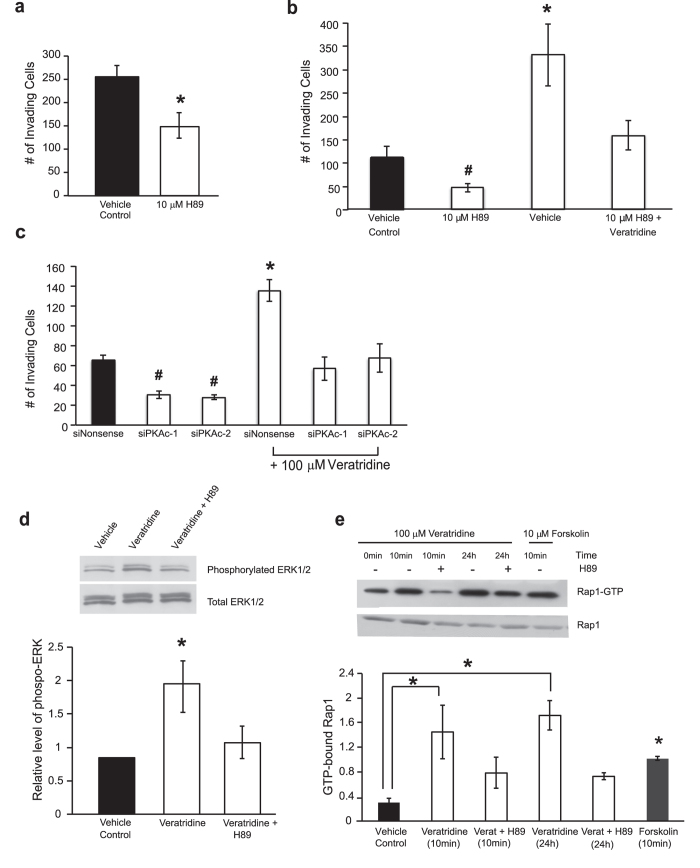
Veratridine signals through PKA to enhance invasiveness and persistent MAPK signaling in SW620 colon cancer cells. **a**) Baseline Matrigel invasion by SW620 cells over 48 hr was significantly decreased in the presence of 10 μM H89, a PKA inhibitor. **b)** Veratridine-stimulated (100 μM) invasion by SW620 cells at 48 hr was inhibited by pretreatment with 10 μM H89. **c)** Veratridine-stimulated (100 μM) invasion by SW620 cells at 48 hr is inhibited by siRNA-mediated knockdown of PKA. Two separate siRNAs targeting both the alpha and beta PKA catalytic subunit isoforms were employed in knockdown experiments (siPKAc-1 = siPKACA_1 + siPKACB_1, and siPKAc-2 = siPKACA_2 + siPKACB_2). **d)** Western blot analysis demonstrated an inhibition of 100 μM veratridine-stimulated ERK1/2 phosphorylation in SW620 cells at 48 hr following 10 μM pretreatment with the PKA inhibitor H89. Representative blot is shown (top). **e)** Veratridine-stimulated Rap1 loading of GTP is inhibited by 10 μM pretreatment with the PKA inhibitor H89. Forskolin (10 μM) served as a positive control. Representative blot is shown (top). Results are means ± SEM from at least 3–6 independent experiments for each time point in a-e. * Significantly different from vehicle control, P < 0.05, two-sided unpaired t-test (a); * significantly different for all pairwise comparisons (b-d), # significantly different for all pairwise comparisons except for siPKAc-1 versus siPKAc-2 (b and c), P < 0.05, ANOVA with post-hoc Holm test, all other pairwise comparisons non-significant (b-d); * significantly different from Vehicle Control, P < 0.05, ANOVA with post-hoc Dunnett’s (e).

Depolarization-induced stimulation of the MAPK pathway involving Rap1 in neuronal cells can be either PKA-dependent or -independent^16^,^28^. Therefore, the participation of PKA in veratridine-stimulated invasivity of colon cancer cells was investigated. We first measured the effects of the PKA inhibitor H89 on the baseline and veratridine-stimulated invasion of SW620 cells. In the presence of 10 μM H89, baseline Matrigel invasion was significantly decreased over 48 hr (Fig. 6a). Likewise, H89 co-treatment impaired the ability of 100 μM veratridine to increase SW620 cell invasion over 48 hr (Fig. 6b). Identical results were observed following targeted knockdown of the PKA catalytic subunit (*PKAc*) by two different siRNAs (Fig. 6c). Again it is noteworthy that knockdown of *PKAc* led to a reversal of veratridine-stimulated invasion back to baseline, but not sub-baseline, levels. In parallel experiments, 48 hr veratridine-induced phosphorylation of ERK1/2 was significantly impaired by the presence of 10 μM H89 (Fig. 6d). Lastly, veratridine-stimulated GTP loading of Rap1 at 10 min and 24 hr was significantly inhibited following inhibition of PKA by 10 μM H89 pretreatment (Fig. 6e). Taken together, these findings suggest that veratridine induces persistent ERK1/2 activation and associated SW620 cell invasion via a Rap1B- and PKA-dependent mechanism.

**Figure 7:**
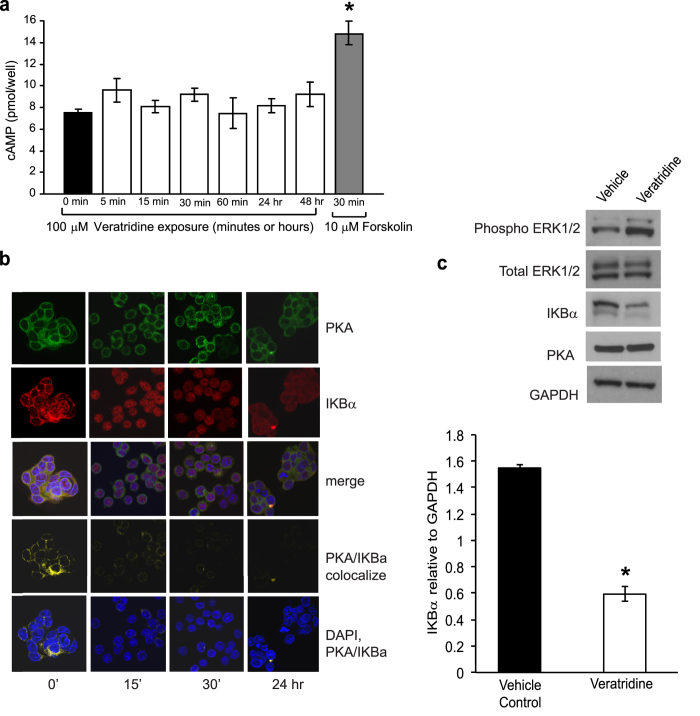
Veratridine does not promote cAMP accumulation but abrogates co-localization of IkBα and PKA. **a)** SW620 cells were grown to confluence in 12 well plates and serum starved for 18 hr prior to treatment with 100 μM veratridine or 10 μM forskolin for the indicated time periods. Veratridine stimulation did not induce accumulation of cAMP (as measured by RIA) compared to control (0 min). Forskolin treatment (positive control) induced a significant increase in cAMP immunoreactivity. Results are means ± SEM from at least 3 independent experiments for each time point. **b)** Veratridine stimulation (100 μM) reverses cytoplasmic colocalization of PKA and IKBα. Confocal immunofluorescence of SW620 cells were triple-stained for PKA catalytic subunit alpha isoform 2 (green), IkBα (red) and nuclei by DAPI (blue). PKA/IkBα colocalization shown in yellow (PKA/IkBα colocalize panels). Confocal images are representative of 6 independent experiments. **c)** Western blot analysis demonstrates veratridine-stimulated ERK1/2 phosphorylation in SW620 cells at 30 min correlates with IkBα degradation. Results are means ± SEM from at least 3 independent experiments, * Significantly different from 0 min control, P < 0.05, ANOVA with post-hoc Dunnett’s (a); * significantly different from Vehicle P < 0.05, two-sided unpaired t-test (c).

It has been shown that VGSC activation can stimulate adenylyl cyclase (AC) activity in a voltage-gated calcium channel (VGCC)-independent manner in neuronal cells^30^,^31^. We have previously demonstrated that VGSC activity stimulates invasion in colon cancer cells in a VGCC-independent manner^4^. Accordingly, we used a radioimmunoassay to test an involvement of elevated cAMP in the effects of 100 μM veratridine on SW620 cells across a range of time points. PKA-dependent invasion of SW620 cells may be due to elevated cAMP levels following veratridine-mediated activation of VGSCs. Cells were grown to confluence and serum starved for 18 hr prior to drug treatment. Veratridine treatment did not significantly effect cAMP production compared to vehicle at any of the time points tested (Fig. 7a). Forskolin, a direct activator of adenylyl cyclase, was used as a positive control and significantly enhanced cAMP production with 30 min exposure. Hence, these findings suggest that PKA-dependent invasion by SW620 cells does not involve accumulation and subsequent binding of cAMP to the PKA regulatory subunit (PKAr), which releases the PKAc subunit from inhibitory control. Besides PKAr, IkBα is a known inhibitor of PKA activity by forming an inhibitory complex with the PKAc subunit^32^. We therefore assessed the affect of veratridine stimulation on colocalization of PKAc with IkBα. Interestingly, veratridine treatment at 15 min, 30 min and 24 hr, abrogated cytoplasmic colocalization of IkBα with PKAc (Fig. 7b). Moreover, western blot analysis demonstrated a significant degradation of IkBα following 30 min veratridine stimulation (Fig. 7c). To determine whether forskolin-activated adenylyl cyclase could further enhance the veratridine-stimulated phosphorylation of ERK1/2, we analyzed phosphorylated ERK levels after 30 min exposure to forskolin, veratridine and in combination and found no significant differences among these treatment groups (Supplemental Fig. S1). These findings confirm a cAMP-independent mechanism for veratridine-stimulated ERK1/2 activation.

**Figure 8:**
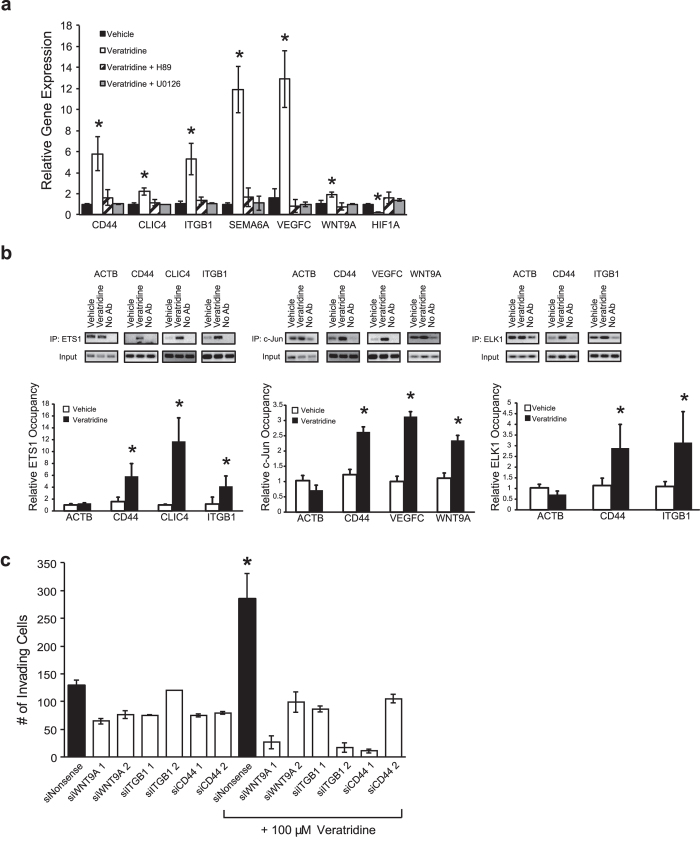
Activation of sodium channels induces expression changes of invasion-related genes via a PKA/ERK/c-JUN/ELK-1/ETS-1 transcriptional pathway. **a)** Veratridine-stimulation of invasion-related gene expression in SW620 cells at 24 hr was reversed by pretreatment with the PKA inhibitor H89 and MEK inhibitor U0126. qRT-PCR results of invasion-related gene expression in SW620 cells treated with vehicle, 100 μM veratridine, 100 μM veratridine +10 μM H89, or 100 μM veratridine +10 μM U0126. **b)** Veratridine increased binding of transcription factors ETS1, c-Jun and ELK1 to invasion-related genes as shown by ChIP-PCR. Representative blots are depicted above bar graphs. ACTB (actin B) served as a negative control where veratridine stimulation did not increase transcription factor binding to the ACTB promoter compared to vehicle-treated cells. IP, immunoprecipitation of transcription factor bound to chromatin; No Ab, no antibody treatment control. **c)** siRNA-mediated knockdown of invasion-related genes abrogated veratridine-stimulated invasion of SW620 cells through Matrigel at 48 hr. Results are means ± SEM from at least 3–6 independent experiments for each time point in a-c. * Significantly different from corresponding vehicle control, P < 0.05, ANOVA with post-hoc Dunnett’s (a); * significantly different from corresponding vehicle control, P < 0.05, two-sided unpaired t-test (b); * significantly different for all pairwise comparisons, P < 0.05, ANOVA with post-hoc Holm test (c).

The combination of persistent activation of the PKA/Rap1B/MEK/ERK pathway resulting from veratridine-induced stimulation of VGSC activity and subsequent increase in invasive potential of SW620 cells (24–72 hr) is highly suggestive of a downstream transcriptional mechanism. A number of TFs are known to be activated by ERK1/2^33^ . Consequently, we evaluated the involvement of the promoter regions of genes previously shown to be transcriptionally regulated following *SCN5A* knockdown^4^. PROMO was used to evaluate DNA sequences for TF binding sites (http://alggen.lsi.upc.es/cgi-bin/promo_v3/promo/promoinit.cgi?dirDB = TF_8.3). The first 5000 bases upstream of the transcription start site were screened for TF binding motifs that correspond to transcription factors regulated by ERK1/2^33^. Several *SCN5A*-regulated genes, including *CD44*, *CLIC4*, *ITGB1*, *SEMA6A*, *VEGFC*, *WNT9A* and *HIF1A* were shown to have motifs within 1000 bases of the transcription start site for TFs regulated by ERK1/2, namely c-JUN, ETS-1, and ELK-1. Treatment of SW620 cells with 100 μM veratridine for 24 hr resulted in a significant 2–12 fold increase in *CD44*, *CLIC4*, *ITGB1*, *SEMA6A*, *VEGFC*, *WNT9A* mRNAs, and a 5-fold decrease in *HIF1A* mRNA (Fig. 8a). The veratridine-stimulated changes in gene expression were dependent on both PKA and ERK1/2, as treatment of SW620 cells with either 10 μM H89 or 10 μM U0126, respectively, abrogated these transcriptional changes (Fig. 8a). We tested a subset of these genes and confirmed that veratridine treatment significantly increased ERK1/2-regulated TF binding to *CD44*, *CLIC4*, *ITGB1*, *VEGFC* and *WNT9A* gene promoters based on ChIP-PCR (Fig. 8b). Finally, we tested siRNA-mediated knockdown of *WNT9A*, *ITGB1* and *CD44* in SW620 cells and demonstrate a reversal of veratridine-stimulated Matrigel invasion (Fig. 8c). Our results define a PKA/Rap1B/MEK/ERK/c-JUN/ELK-1/ETS-1 transcriptional pathway that is activated by VGSCs leading to increased invasive potential.

## Discussion

Increasing evidence suggests that ion channels are key contributors to the oncogenic behavior of cancer cells^13^. We have previously demonstrated that the Na_V_1.5 VGSC contributes to the invasion potential of colon cancer cells and regulates an invasion-related gene network for colon cancer^4^. The current study sought to elucidate the signaling pathway by which these channels regulate gene expression changes important for oncogenic potential.

It has been proposed that VGSC activity increases invasiveness of cancer cells by facilitating activation of specific proteases or by inducing podosome formation^9^,^12^. Although these mechanisms might apply to colon cancer as well, we chose to evaluate transcriptional changes that might occur as a result of Na_V_1.5 expression and activation. There have been no systematic studies examining VGSC activity together with changes in gene expression and invasion potential of cancer cells. Ion channel activity stimulates a variety of intracellular signaling pathways and the functional state of ion channels is known to affect gene expression in neuronal and skeletal muscle cells^34^,^35^,^36^.

In an effort to understand cancer signaling mechanism(s) connecting VGSC activity to transcriptional changes in invasion-related genes, we tested a model based on the known signaling consequences of VGSC activation in excitable cells in which voltage-gated ion channels are normally expressed. Given that many neuronal genes are usurped during colon cancer progression, it is possible that pathways activated by depolarization in excitable cells may also be activated in cancer cells. The MAPK pathway is a well-established signal transduction pathway deregulated in about 1/3 of all cancers and coincidentally is activated in response to depolarization of neurons^14^,^16^. Upon activation, the ERK1/2 MAPKs can activate various TFs including ELK1, ETS-1, and c-JUN, among others^14^,^37^. The promoter regions of genes directly regulated by Na_V_1.5 knockdown in colon cancer cell lines were analyzed in the present study and determined to have overrepresented binding motifs corresponding to ERK-regulated TFs. This study confirmed that pharmacological manipulation of VGSC activity led to changes in both ERK1/2 activity and chromatin binding of ERK-regulated TFs to *SCN5A*-dependent genes. These observations led us to postulate that Na_V_1.5 VGSCs signal through the ERK MAPK pathway to transcriptionally regulate genes that drive colon cancer invasion.

Despite expression of VGSCs and other genes associated with excitable cells, SW620 colon cancer cells appear not to be electrically excitable. They have a depolarized membrane potential of approximately −40 mV^22^. A comparison of the voltage-dependence of current activation with that of steady-state inactivation reveals that there is a window of available current remaining at −40 mV^4^. While Na_V_1.5 channels are predominantly in the inactivated state at this potential they briefly visit the active conformation allowing some persistent Na^+^ entry. Blockade of Na_V_1.5 VGSCs by lidocaine at concentrations within the micromolar range occurs through high affinity use-dependent binding that requires depolarization^38^ . Through this mechanism lidocaine preferentially blocks channels in the open and inactivated states and provides an opportunity for selective inhibition of Na^+^ entry into VGSCs of colon cancer cells without affecting Na^+^ entry into cardiac cells. Conversely, veratridine enhances steady-state current through Na_V_1.5 VGSCs of SW620 cells resulting in reduced inactivation. This results in a large increase in the availability of steady-state current through VGSCs at depolarized membrane potentials. Similar findings have previously been reported for the effects of veratridine on Na_V_1.6 VGSCs^39^.

Pharmacological and genetic tools were used to probe changes in invasiveness and ERK1/2 activity in colon cancer cells to identify additional participating signaling molecules. Although activation of VGCCs is known to occur following depolarization of neurons to stimulate MAPK^16^, our previous work suggested a VGCC-independent mechanism^4^. Furthermore, it has been demonstrated that Na^+^ can directly activate AC to stimulate cAMP production^30^. However, we observed that VGSC activation did not lead to any appreciable cAMP accumulation in SW620 colon cancer cells. These findings suggest a VGSC signaling mechanism leading to MAPK activation that is, in this respect, distinct from that observed in excitable cells.

Our study demonstrates that persistent activation of ERK1/2 MAPKs following veratridine stimulation is PKA-dependent in colon cancer cells. This is an intriguing finding, given that PKA activity is important for depolarization-induced signaling to the MAPKs in neurons^16^,^29^,^40^. In addition, our study suggests that VGSCs signal through PKA in a cAMP-independent manner to enhance ERK1/2 activity and invasion of colon cancer cells. A number of studies have demonstrated cAMP-independent activation of PKA^41^,^42^,^43^,^44^. For example, a cytosolic pool of PKAc can associate with NF-kB p65 and I kBα as part of an inhibitory complex^32^. Following signaling events leading to phosphorylation of I kBα, the inhibitory complex dissociates, leading to proteasomal degradation of I kBα and the freeing of PKAc. This appears to be the likely mechanism in SW620 cells as veratridine stimulation abrogates colocalization of PKAc and I kBα and induces IkBα degradation with a time course that induces both Rap1B and ERK1/2 activation. Alternative cAMP-independent mechanisms for induction of PKA activity may include stimulation by sphinogsine, a signaling intermediate of the sphingomyelin pathway^41^,^42^. Future studies will clarify VGSC-activated signaling events leading to phosphorylation of NF-kB p65 and PKAc release, and whether additional cAMP-independent mechanisms may be involved in PKA activation in colon cancer cells.

In addition to PKA the involvement of the small G protein Rap1 in depolarization-induced signaling to MAPK has been well-characterized^16^,^28^,^29^,^40^. Activation of Rap1A promotes invasive and metastatic potential of prostate cancer cell lines^23^, including those cell lines shown to express VGSCs^43^. However, a link between VGSC activity and Rap1 stimulation in mediating prostate cancer cell invasion/metastasis has not been established. Thus, we investigated the participation of Rap1 in the VGSC signaling in colon cancer cells and found that VGSCs signal through both Rap1A and Rap1B to increase invasion potential. Rap1 activation occurred within 5 min of veratridine treatment and persisted for at least 24 hr in a PKA-dependent manner. The data further suggest that although VGSCs signal through both Rap1A and Rap1B in colon cancer cells, Rap1B activation in particular leads to persistent ERK1/2 MAPK activation. It is possible that upon activation, Rap1A signals through an integrin-dependent mechanism, which has been shown for prostate cancer cells^26^, while Rap1B signals to MAPK to enhance colon cancer cell invasion. This possibility is further supported by the fact that Rap1B is phosphorylated and activated by PKA^44^, and VGSC signaling to ERK1/2 requires PKA and Rap1B in colon cancer cells (this study).

Taken together, our inhibitor and knockdown experiments suggest that veratridine-stimulated invasion by SW620 colon cancer cells is mediated by an *SCN5A*-dependent pathway involving PKA/RAP1B/MEK/ERK (predominant pathway). As inhibitor and knockdown treatments reversed veratridine-stimulated invasion to baseline but not sub-baseline levels (i.e. inhibitor or knockdowns in the absence of veratridine), a second minor *SCN5A*-independent pathway may also be present. The nature of this minor component remains to be elucidated, but may involve K^+^ and/or Ca^++^ channels that are known to be stimulated by veratridine in a TTX-independent fashion^45^,^46^. Finally, this study along with our previous findings strongly support the notion that Na_V_1.5 is the primary VGSC subunit responsible for veratridine-stimulated ERK1/2 phosphorylation and corresponding invasive activity in SW620 colon cancer cells. First, knockdown of *SCN5A* inhibits veratridine-stimulated phosphorylation of ERK1/2 back to baseline levels; and second, *SCN5A* mRNA is the major VGSC mRNA species expressed in SW620 cells^4^.

In conclusion, we provide evidence for VGSCs in colon cancer cells signaling through a novel pathway that is VGCC-independent, cAMP-independent and involves PKA, Rap1B, MEK and persistent activation ERK1/2, leading ultimately to transcriptional induction of invasion-related genes. These important data highlight the contribution of VGSCs to the metastatic potential of colon cancer cells and suggest that pharmacological manipulation of channel activity may provide clinical benefit. Given the broad toxicity of current therapies, a more targeted approach blocking VGSC activity may provide a better tolerated alternative treatment.

Recent mouse xenograft studies with human breast cancer cells demonstrate that the VGSC inhibitors, phenytoin, an antiepileptic drug, and the antiarrhythmic agent ranolazine, inhibit invasion and metastasis *in vivo*^47^,^48^ . It may be beneficial to repurpose such drugs for use in the treatment of breast cancer. Our study further suggests that administration of local anesthetics during surgical resection should be explored as a potential therapeutic strategy for the prevention of metastases. There is an urgent need for the development of drugs that inhibit metastasis given that the majority of the classic cytotoxic chemotherapeutic drugs in current use only target cell proliferation. In light of our work in colon cancer, studies identifying a role for VGSCs in the progression of other human cancers and the typically large therapeutic window characteristic of many VGSC modulators, it is worthwhile to further explore the feasibility of VGSC modulators in cancer therapy.

## Methods

*Cell Culture*- SW620 human colon cancer cells (Catalog No. CCL-227) were obtained directly from American Type Culture Collection (ATCC). Cells were maintained in Dulbecco’s Modified Eagle Medium (DMEM) supplemented with 10% heat inactivated fetal bovine serum (FBS), 2 mM L-glutamine and penicillin-streptomycin (Gibco, Grand Island, NY) at 37 °C and 5% CO_2_. All experiments were performed using cells passaged less than ten times.

*RNA Extraction and Quantitative RT-PCR*- Total RNA was isolated from cells using Trizol (Invitrogen, Carlsbad, CA) extraction according to manufacturer’s instructions. Crude RNA was purified using RNeasy Mini Kit (Qiagen, Valencia, CA) as described by the manufacturer and treated with DNase to remove contaminating DNA. The final concentration of RNA was determined using a NanoDrop spectrophotometer (NanoDrop Technologies, Inc., Wilmington, DC). RNA integrity was confirmed using the 260/280 absorbance ratio and agarose gel electrophoresis. RNA was stored at −80 °C. Total purified RNA was reverse transcribed with random primers using Taqman reverse transcription reagents (Applied Biosystems, Carlsbad, CA). Resulting cDNA was used as a template for qRT-PCR. PCR primers were designed using Primer3 and selected for specificity using the NCBI BLAST tool. Analysis of gene expression was performed on an ABI Prism 7300 Sequence Detection System using SYBR green (Applied Biosystems, Carlsbad, CA). Amplicon specificity was verified by first-derivative melting curve analysis. Housekeeping genes, pyrophosphatase (inorganic) 1 (PPA1) and eukaryotic translation initiation factor 1 A, X-linked (EIFAX) (Genbank Accession number NM_021129 and NM_001412, respectively), were used for normalization. Quantitation and normalization of relative gene expression were accomplished using the comparative threshold cycle method or ΔΔC_T_. Primer sequences are provided in Supplemental Table 1.

*Electrophysiology*- Cells were cultured for 48 hr to 30% confluence in 35-mm dishes for electrophysiological studies. The whole-cell patch-clamp technique was used to record voltage-activated currents from individual cells. The electrode solution contained 130 mM CsCl, 15 mM NaCl, 2 mM MgCl_2_, 10 mM EGTA, and 10 mM 4-(2-hydroxyethyl)-1-piperazineethanesulfonic acid (HEPES) (pH 7.4). The extracellular solution used for recording Na^+^ currents contained 140 mM NaCl, 4.7 mM KCl, 1.2 mM MgCl_2_, 2.5 mM CaCl_2_, 10 mM HEPES, and 11 mM glucose (pH 7.4) with and without the indicated concentrations of lidocaine. Veratridine was diluted in extracellular solution and bath applied. Currents were recorded using an Axopatch 200 B amplifier, low-pass filtered at 5 KHz, digitized at 10 KHz using a Digidata 1320 A interface, acquired using pCLAMP8 software, and analyzed using Clampfit 10.1 software (all from Molecular Devices, Sunnyvale, CA). Current-voltage relationships were recorded with depolarizing steps from −80 mV to +70 mV in 10 mV steps. Steady state inactivation data were recorded from cells exposed to 100 ms pre-pulses to voltages between -140 mV and −10 mV before activation to 0 mV. Activation and inactivation curves were derived and fitted with a Boltzmann function as described previously^22^. The logistic equation was used to fit data and determine IC_50_ values for lidocaine.

*siRNA Transfections*- Cells were cultured for 24 hr to 50% confluence before transfection with Dharmacon On-Target*plus* short interfering RNA (siRNA) duplexes according to manufacturer’s instructions (Thermo Fisher Scientific, Lafayette, CO). Briefly, cells were transfected with Dharmafect 4 transfection reagent and individual siRNAs at a final concentration of 1% v/v and 100 nM, respectively. Cells were maintained in the presence of transfection reagent under normal culture conditions for 24–48 hr before being used in qRT-PCR, western blot analysis, and Matrigel experiments. Two different siRNAs were employed for each gene knockdown and knockdown efficiency, ranging from 60–90% compared to nonsense siRNA control, was confirmed for all assays (i.e. gene expression, western blot and Matrigel) by qRT-PCR (Supplemental Fig. S2). siRNA sequences are provided in Supplemental Table 1.

*Western Blots*- Cells were grown to 80% confluence and lysates collected using RIPA lysis buffer (Santa Cruz Biotechnology, Santa Cruz, CA). Protein concentration was determined using NanoOrange Protein Quantitation Kit (Molecular Probes, Invitrogen, Eugene, OR). Fifty μg of protein was separated using standard SDS-PAGE with precast 4–20% gels (BioRad, Hercules, CA) and transferred to PVDF membranes using standard transfer conditions. Transfer conditions for Rap1 blots was overnight at 20 V and all other protein blots were 1 hour at 100 V. Membranes were washed with TBST and blocked for at least one hour using 5% BSA or 5% powdered milk (Rap1 westerns). The Na_V_1.5 antibody is goat polyclonal purchased from Santa Cruz Biotechnology. All remaining antibodies used in this study are rabbit polyclonal, purchased from the following distributors: phospho-ERK1/2 (Cell Signaling, Danvers, MA), IKBα, Erk1 and Rap1 (Santa Cruz, Technologies, Santa Cruz, CA), GAPDH (mouse) (Millipore, Temecula, CA). Membranes were incubated with primary antibodies diluted in 5% BSA for at least 2 hr at 4 °C. Membranes were washed 3–4 times using TBST. Membranes were incubated with HRP-conjugated secondary antibody (Santa Cruz Biotechnology) diluted in 5% milk for 1 hour at room temperature. After four washes, membranes were incubated with enhanced chemiluminescence substrate (Perkin Elmer, Waltham, MA) for 5 min and exposed to autoradiography film for 1 min. Films were scanned using a Personal Densitometer SI (Molecular Dynamics, Sunnyvale, CA) and protein bands quantified using ImageQuant software. Membranes were incubated in stripping buffer (2% SDS, 62.5 mM Tris-HCl, 100 mM β-mercaptoethanol) for 15 min at 50 °C to allow stripping of initial antibodies. Membranes were then incubated with control antibodies for quantification and normalization.

*Matrigel Invasion Assays*- Cells (3 × 10^5^ or 6 × 10^5^ for stimulatory or inhibitory studies, respectively) were seeded in the top well of a Matrigel-coated invasion chamber (BD Biosciences) in DMEM containing 0.1% serum with or without pharmacological agents. The bottom well was filled with 750 μL DMEM containing 10% serum as a chemoattractant with or without pharmacological agents. After 24–72 hr, non-invading cells were scraped from the upper side of the insert using a cotton swab. Invading cells on the bottom of the insert were fixed and stained with Diff-Quick Stain (IMEB, Inc., San Marcos, CA) according to manufacturer’s instructions. The total number of invading cells was counted for each insert under a light microscope.

*cAMP Radioimmunoassay*- Cells were grown to confluence in 12-well plates and serum-starved for 18 hr. Cells were pretreated with 1 mM 3-isobutyl-1-methylxanthine (IBMX) for 20 min to inhibit cAMP phosphodiesterases, followed by agonist treatment for the indicated times at 37 °C. Cold ethanol was used to extract cAMP from the cells. The resulting pellet was resuspended in 200 μl assay buffer and cAMP levels were detected in duplicate using a ^125^I radioimmunoassay kit according to manufacturer’s instructions including the acetylation procedure (Perkin Elmer, Waltham, MA). Samples were counted using a one minute counting time in a Packard Cobra II gamma counter. A standard curve was generated in Microsoft Excel and counts were converted to pmol per well.

*Rap1-GTP Pull-Down Assays*- The cDNA of the effector of Rap1 (Rap1 binding domain of RalGDS) cloned into BL21 *Escherichia coli* cells was a generous gift from Atsuko Sakurai. Bead slurries were prepared as previously described^49^ and stored at 4 °C until ready to use. SW620 cells were grown in 10 cm plates to 70% confluence and serum starved for 18 hr before treating with drugs at indicated time points. The cells were washed with cold PBS and lysed using lysis buffer with protease inhibitors (20 mM HEPES (pH 7.5), 1% Triton X–100, 100 mM NaCl, 20 mM MgCl_2_, EGTA, β-glycerol phosphate, 1 mM PMSF, 10 μg/ml aprotinin and leupeptin, 1 mM Na_3_VO_4_, 1 mM Dithiothreitol (DTT), and water). Lysates were centrifuged at 13,200 rpm at 4 °C for 10 min. For total cell lysates, 50 μl precleared lysates were immediately placed in 50 μl 2X Laemmli sample buffer and stored at 4 °C. For pull down lysates, 450 μl precleared lysates were incubated with 20 μl GST-RalGDS bead slurry at 4 °C with rotation for 30 min. Pull down lysates were then centrifuged, washed with lysis buffer, and resuspended in 50 μl 2X Laemmli sample buffer and stored at 4 °C. Pull down and total cell lysates were then subjected to standard SDS-PAGE.

*Chromatin Immunoprecipitation-PCR (ChIP-PCR) Assays-* ChIP Assay Kit was purchased from Millipore Corp., and assays were performed according to the manufacturer’s instructions and as previously described^50^. Antibodies for EST1 (ab10936), c-JUN (ab31419), and ELK1 (ab28831) were from Abcam (Cambridge, MA). The quantification of transcription factor binding to target genes was calculated by measuring the ratio of ChIP-to-Input, and the non-antibody treated ChIP sample served as a negative control. All primers used for ChIP-PCRs are listed in Supplemental Table 1.

*Immunocytochemistry (ICC)-* ICC in SW620 cells was performed as previously described^51^. Antibodies for PKA (catalytic subunit alpha isoform 2, ab26322)) and IKBα (sc-1643) were purchased from Abcam (Cambridge, MA) and Santa Cruz Biotechnology (Santa Cruz, CA), respectively.

## Additional Information

**How to cite this article**: House, C. D. *et al.* Voltage-gated Na^+^ Channel Activity Increases Colon Cancer Transcriptional Activity and Invasion Via Persistent MAPK Signaling. *Sci. Rep.* **5**, 11541; doi: 10.1038/srep11541 (2015).

## Supplementary Material

Supplementary Information

## References

[b1] Hodgkin A. L. & Huxley A. F. A quantitative description of membrane current and its application to conduction and excitation in nerve. The Journal of physiology 117, 500–44 (1952). 12991237 10.1113/jphysiol.1952.sp004764 PMC1392413

[b2] Roger S. *et al.* Voltage-gated sodium channels potentiate the invasive capacities of human non-small-cell lung cancer cell lines. The international journal of biochemistry & cell biology 39, 774–86 (2007). 17307016 10.1016/j.biocel.2006.12.007

[b3] Brackenbury W. J. & Djamgoz M. B. Activity-dependent regulation of voltage-gated Na+ channel expression in Mat-LyLu rat prostate cancer cell line. The Journal of physiology 573, 343–56 (2006). 16543264 10.1113/jphysiol.2006.106906 PMC1779734

[b4] House C. D. *et al.* Voltage-Gated Na+ Channel SCN5A Is a Key Regulator of a Gene Transcriptional Network That Controls Colon Cancer Invasion. Cancer Res 70, 6957–67 (2010). 20651255 10.1158/0008-5472.CAN-10-1169 PMC2936697

[b5] Bennett E. S., Smith B. A. & Harper J. M. Voltage-gated Na+ channels confer invasive properties on human prostate cancer cells. Pflugers Arch 447, 908–14 (2004). 14677067 10.1007/s00424-003-1205-x

[b6] Laniado M. E. *et al.* Expression and functional analysis of voltage-activated Na+ channels in human prostate cancer cell lines and their contribution to invasion *in vitro*. The American journal of pathology 150, 1213–21 (1997). 9094978 PMC1858184

[b7] Fraser S. P. *et al.* Voltage-gated sodium channel expression and potentiation of human breast cancer metastasis. Clin Cancer Res 11, 5381–9 (2005). 16061851 10.1158/1078-0432.CCR-05-0327

[b8] Onkal R. & Djamgoz M. B. Molecular pharmacology of voltage-gated sodium channel expression in metastatic disease: clinical potential of neonatal Nav1.5 in breast cancer. Eur. J. Pharmacol. 625, 206–219 (2009). 19835862 10.1016/j.ejphar.2009.08.040

[b9] Gillet L. *et al.* Voltage-gated Sodium Channel Activity Promotes Cysteine Cathepsin-dependent Invasiveness and Colony Growth of Human Cancer Cells. The Journal of biological chemistry 284, 8680–91 (2009). 19176528 10.1074/jbc.M806891200 PMC2659227

[b10] Brisson L. *et al.* Na(V)1.5 enhances breast cancer cell invasiveness by increasing NHE1-dependent H(+) efflux in caveolae. Oncogene 30, 2070–2076 (2011). 21170089 10.1038/onc.2010.574

[b11] Brisson L. *et al.* NaV1.5 Na(+) channels allosterically regulate the NHE-1 exchanger and promote the activity of breast cancer cell invadopodia. J. Cell. Sci. 126, 4835–4842 (2013). 23902689 10.1242/jcs.123901

[b12] Carrithers M. D. *et al.* Regulation of podosome formation in macrophages by a splice variant of the sodium channel SCN8A. The Journal of biological chemistry 284, 8114–26 (2009). 19136557 10.1074/jbc.M801892200 PMC2658105

[b13] Yang M. & Brackenbury W. J. Membrane potential and cancer progression. Front. Physiol. 4, 185 (2013). 23882223 10.3389/fphys.2013.00185 PMC3713347

[b14] Dhillon A. S., Hagan S., Rath O. & Kolch W. MAP kinase signalling pathways in cancer. Oncogene 26, 3279–90 (2007). 17496922 10.1038/sj.onc.1210421

[b15] Fang J. Y. & Richardson B. C. The MAPK signalling pathways and colorectal cancer. Lancet Oncol. 6, 322–327 (2005). 15863380 10.1016/S1470-2045(05)70168-6

[b16] Baldassa S., Zippel R. & Sturani E. Depolarization-induced signaling to Ras, Rap1 and MAPKs in cortical neurons. Brain research 119, 111–22 (2003). 14597235 10.1016/j.molbrainres.2003.08.020

[b17] Rosen L. B., Ginty D. D., Weber M. J. & Greenberg M. E. Membrane depolarization and calcium influx stimulate MEK and MAP kinase via activation of Ras. Neuron 12, 1207–21 (1994). 8011335 10.1016/0896-6273(94)90438-3

[b18] Perez-Gomez A. & Tasker R. A. Transient domoic acid excitotoxicity increases BDNF expression and activates both MEK- and PKA-dependent neurogenesis in organotypic hippocampal slices. BMC Neurosci. 14, 72-2202–14-72 (2013). 23865384 10.1186/1471-2202-14-72 PMC3722092

[b19] Agell N., Bachs O., Rocamora N. & Villalonga P. Modulation of the Ras/Raf/MEK/ERK pathway by Ca(2+), and calmodulin. Cell. Signal. 14, 649–654 (2002). 12020764 10.1016/s0898-6568(02)00007-4

[b20] Roskoski R. Jr. ERK1/2 MAP kinases: structure, function, and regulation. Pharmacol. Res. 66, 105–143 (2012). 22569528 10.1016/j.phrs.2012.04.005

[b21] Campbell T. M., Main M. J. & Fitzgerald E. M. Functional expression of the voltage-gated Na(+)-channel Nav1.7 is necessary for EGF-mediated invasion in human non-small cell lung cancer cells. J. Cell. Sci. 126, 4939–4949 (2013). 23986482 10.1242/jcs.130013

[b22] Baptista-Hon D. T. *et al.* Potent inhibition by ropivacaine of metastatic colon cancer SW620 cell invasion and NaV1.5 channel function. Br. J. Anaesth. 113 Suppl 1, i39–i48 (2014). 24852501 10.1093/bja/aeu104

[b23] Andrikopoulos P. *et al.* Angiogenic Functions of Voltage-gated Na+ Channels in Human Endothelial Cells: MODULATION OF VASCULAR ENDOTHELIAL GROWTH FACTOR (VEGF) SIGNALING. J. Biol. Chem. 286, 16846–16860 (2011). 21385874 10.1074/jbc.M110.187559 PMC3089528

[b24] Okura D. *et al.* Lidocaine preferentially inhibits the function of purinergic P2×7 receptors expressed in Xenopus oocytes. Anesth. Analg. 120, 597–605 (2015). 25695577 10.1213/ANE.0000000000000585

[b25] Roger S. *et al.* Understanding the roles of the P2×7 receptor in solid tumour progression and therapeutic perspectives. Biochim. Biophys. Acta DOI: 10.1016/j.bbamem.2014.10.029 (2014). 10.1016/j.bbamem.2014.10.029 25450340

[b26] Bailey C. L., Kelly P. & Casey P. J. Activation of Rap1 promotes prostate cancer metastasis. Cancer Res. 69, 4962–4968 (2009). 19470770 10.1158/0008-5472.CAN-08-4269 PMC4195850

[b27] Dao V. T., Dupuy A. G., Gavet O., Caron E. & de Gunzburg J. Dynamic changes in Rap1 activity are required for cell retraction and spreading during mitosis. J. Cell. Sci. 122, 2996–3004 (2009). 19638416 10.1242/jcs.041301

[b28] Bouschet T. *et al.* Stimulation of the ERK pathway by GTP-loaded Rap1 requires the concomitant activation of Ras, protein kinase C, and protein kinase A in neuronal cells. J. Biol. Chem. 278, 4778–4785 (2003). 12473665 10.1074/jbc.M204652200

[b29] Obara Y., Horgan A. M. & Stork P. J. The requirement of Ras and Rap1 for the activation of ERKs by cAMP, PACAP, and KCl in cerebellar granule cells. J. Neurochem. 101, 470–482 (2007). 17254020 10.1111/j.1471-4159.2006.04390.x

[b30] Cooper D. M., Schell M. J., Thorn P. & Irvine R. F. Regulation of adenylyl cyclase by membrane potential. The Journal of biological chemistry 273, 27703–7 (1998). 9765307 10.1074/jbc.273.42.27703

[b31] Duman R. S., Terwilliger R. Z., Nestler E. J. & Tallman J. F. Sodium and potassium regulation of guanine nucleotide-stimulated adenylate cyclase in brain. Biochem. Pharmacol. 38, 1909–1914 (1989). 2545204 10.1016/0006-2952(89)90488-7

[b32] Zhong H., SuYang H., Erdjument-Bromage H., Tempst P. & Ghosh S. The transcriptional activity of NF-kappaB is regulated by the IkappaB-associated PKAc subunit through a cyclic AMP-independent mechanism. Cell 89, 413–424 (1997). 9150141 10.1016/s0092-8674(00)80222-6

[b33] Yoon S. & Seger R. The extracellular signal-regulated kinase: multiple substrates regulate diverse cellular functions. Growth Factors 24, 21–44 (2006). 16393692 10.1080/02699050500284218

[b34] Jull B. A., Plummer H. K. 3rd & Schuller H. M. Nicotinic receptor-mediated activation by the tobacco-specific nitrosamine NNK of a Raf-1/MAP kinase pathway, resulting in phosphorylation of c-myc in human small cell lung carcinoma cells and pulmonary neuroendocrine cells. Journal of cancer research and clinical oncology 127, 707–17 (2001). 11768610 10.1007/s004320100289 PMC12164663

[b35] Roy R., Wewer U. M., Zurakowski D., Pories S. E. & Moses M. A. ADAM 12 cleaves extracellular matrix proteins and correlates with cancer status and stage. The Journal of biological chemistry 279, 51323–30 (2004). 15381692 10.1074/jbc.M409565200

[b36] Juretic N., Urzua U., Munroe D. J., Jaimovich E. & Riveros N. Differential gene expression in skeletal muscle cells after membrane depolarization. Journal of cellular physiology 210, 819–30 (2007). 17146758 10.1002/jcp.20902

[b37] Liu W. H. & Chang L. S. Piceatannol induces Fas and FasL up-regulation in human leukemia U937 cells via Ca2+/p38alpha MAPK-mediated activation of c-Jun and ATF-2 pathways. Int. J. Biochem. Cell Biol. 42, 1498–1506 (2010). 20580678 10.1016/j.biocel.2010.05.007

[b38] Hanck D. A. *et al.* Using lidocaine and benzocaine to link sodium channel molecular conformations to state-dependent antiarrhythmic drug affinity. Circ. Res. 105, 492–499 (2009). 19661462 10.1161/CIRCRESAHA.109.198572 PMC2735213

[b39] Zhu H. L. *et al.* Actions of veratridine on tetrodotoxin-sensitive voltage-gated Na currents, Na1.6, in murine vas deferens myocytes. Br. J. Pharmacol. 157, 1483–1493 (2009). 19552689 10.1111/j.1476-5381.2009.00301.x PMC2765308

[b40] Obara Y., Labudda K., Dillon T. J. & Stork P. J. PKA phosphorylation of Src mediates Rap1 activation in NGF and cAMP signaling in PC12 cells. J. Cell. Sci. 117, 6085–6094 (2004). 15546918 10.1242/jcs.01527

[b41] Cifone M. G. *et al.* Apoptotic signaling through CD95 (Fas/Apo-1) activates an acidic sphingomyelinase. J. Exp. Med. 180, 1547–1552 (1994). 7523573 10.1084/jem.180.4.1547 PMC2191710

[b42] Ma Y. *et al.* Sphingosine activates protein kinase A type II by a novel cAMP-independent mechanism. J. Biol. Chem. 280, 26011–26017 (2005). 15883165 10.1074/jbc.M409081200

[b43] Abdul M. & Hoosein N. Voltage-gated sodium ion channels in prostate cancer: expression and activity. Anticancer Res. 22, 1727–1730 (2002). 12168861

[b44] Hata Y. *et al.* Enhancement of the actions of smg p21 GDP/GTP exchange protein by the protein kinase A-catalyzed phosphorylation of smg p21. J. Biol. Chem. 266, 6571–6577 (1991). 1901063

[b45] Romey G. & Lazdunski M. Lipid-soluble toxins thought to be specific for Na+ channels block Ca2+ channels in neuronal cells. Nature 297, 79–78 (1982). 6280075 10.1038/297079a0

[b46] Verheugen J. A., Oortgiesen M. & Vijverberg H. P. Veratridine blocks voltage-gated potassium current in human T lymphocytes and in mouse neuroblastoma cells. J. Membr. Biol. 137, 205–214 (1994). 8182730 10.1007/BF00232589

[b47] Nelson M., Yang M., Dowle A. A., Thomas J. R. & Brackenbury W. J. The sodium channel-blocking antiepileptic drug phenytoin inhibits breast tumour growth and metastasis. Mol. Cancer. 14, 13 (2015). 25623198 10.1186/s12943-014-0277-x PMC4320839

[b48] Driffort V. *et al.* Ranolazine inhibits NaV1.5-mediated breast cancer cell invasiveness and lung colonization. Mol. Cancer. 13, 264-4598–13-264 (2014). 25496128 10.1186/1476-4598-13-264 PMC4295566

[b49] Sakurai A. *et al.* Semaphorin 3E initiates antiangiogenic signaling through plexin D1 by regulating Arf6 and R-Ras. Mol. Cell. Biol. 30, 3086–3098 (2010). 20385769 10.1128/MCB.01652-09 PMC2876686

[b50] Wang B. D. *et al.* Androgen receptor-target genes in african american prostate cancer disparities. Prostate Cancer. 2013, 763569 (2013). 23365759 10.1155/2013/763569 PMC3556896

[b51] Wang B. D. *et al.* Prostate apoptosis response protein 4 sensitizes human colon cancer cells to chemotherapeutic 5-FU through mediation of an NF kappaB and microRNA network. Mol. Cancer. 9, 98-4598–9-98 (2010). 20433755 10.1186/1476-4598-9-98 PMC2883962

