# Supplementary material for: Voltage-gated Na+ Channel Activity Increases Colon Cancer Transcriptional Activity and Invasion Via Persistent MAPK Signaling
Source: Sci Rep. 2015 Jun 22;5:11541. doi: 10.1038/srep11541 (PMC4476109; doi:10.1038/srep11541)
Supplement: Supplementary Information [file srep11541-s1.pdf]

SUPPLEMENTARY INFORMATION

Voltage-gated Na^+ Channel Activity Increases Colon Cancer Transcriptional Activity and Invasion Via Persistent MAPK Signaling

Carrie D. House[1,4], Bi-Dar Wang[1], Kristin Cennicola[1], Russell Williams[1], May Samaan[2], Jacqueline Olender[1], Vyomesh Patel[2], Daniel T. Baptista-Hon[3], Christina M. Annunziata[4], J. Silvio Gutkind[2], Tim Hales[3] and Norman H. Lee[1*]

[1]Department of Pharmacology and Physiology, The George Washington University, Washington, DC; [2]The National Institute of Dental and Craniofacial Research, Bethesda, MD; [3]The Institute of Academic Anaesthesia, Division of Neuroscience, Ninewells Hospital, University of Dundee, Dundee, DD1 9SY, UK; [4]Women's Malignancies Branch, National Cancer Institute, Bethesda, MD

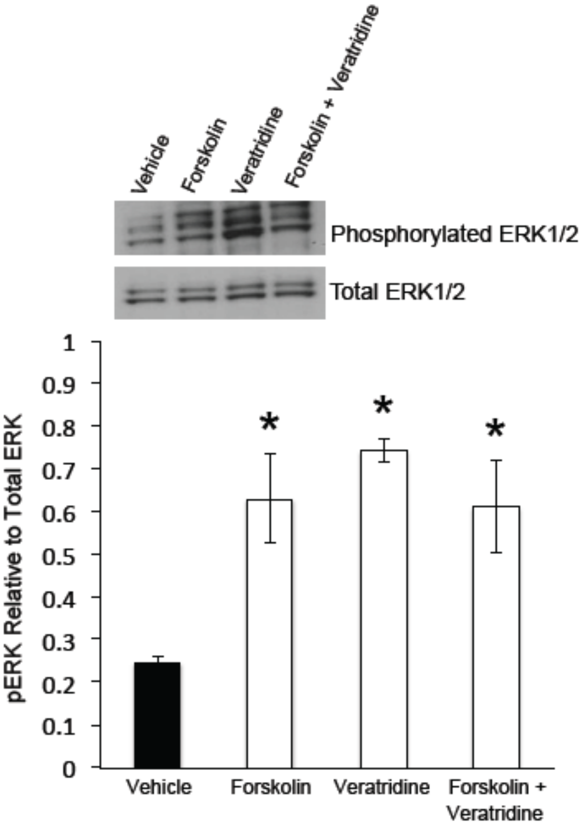

Supplemental Figure S1. ERK1/2 activation is not further increased when adenylyl cyclase is fully activated. SW620 cells were serum starved for 24 hr prior to treatment with 10 μM forskolin in the presence and absence of 100 μM veratridine for 30 min. *Significantly different from vehicle control, $P<0.05$, ANOVA with post-hoc Holm test. All other pairwise comparisons non-significant.

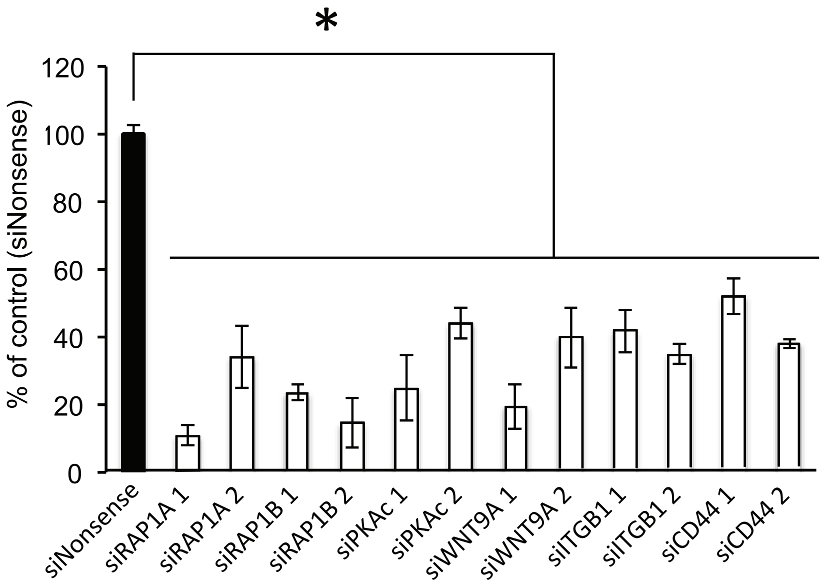

Supplemental Figure S2. mRNA expression levels are significantly reduced after siRNA-mediated knockdown. Two different siRNAs were employed for each gene knockdown with efficiency ranging from 60-90% compared to nonsense siRNA control. Quantitation and normalization of relative gene expression were accomplished using the comparative threshold cycle method or $\Delta\Delta C_T$. Primer sequences are provided in **Supplemental Table 1.** *Significantly different from siNonsense control, $P<0.05$, ANOVA with post-hoc Dunnett's test.

Supplemental Table 1

**RT-PCR Primer sequences**	
EIF1AX_F	GTACTGGAGAGGGGAGAGCA
EIF1AX_R	TGAAGCTGAAACAAGCAGGA
PPA1_F	TGACTTTCCAGTCGGTTTCC
PPA1_R	GGCTGTTGTGGTGACAATGA
CD44_F	GCAGCCCCGATTATTTACAG
CD44_R	GAAGGACACACCCAAGCAAG
CLIC4_F	GTGGCAACTTGGGATTCATT
CLIC4_R	TTCCTCTTGTTAGCCCTCCA
ITGB1_F	AAAGCCCAGAGGCTCCAAAG
ITGB1_R	CCCCTGATCTTAATCGCAAA
SEMA6_F	TACCCACCAGACCAAGAAGG
SEMA6_R	GCAAACCTTCACCAAACCAC
VEGFC_F	GGAGGAGCAGTTACGGTCTG
VEGFC_R	TTGAGGTTGGCCTGTTCTCT
Wnt9A_F	CAAGGATCTGCGAGCCCGTGT
Wnt9A_R	ATGGAAAGGCGCCAACTGCC
HIF1A_F	CAGCAGCCAGACGATCATGCA
HIF1A_R	ACCACGTACTGCTGGCAAAGC
Rap1A_F	AAGTGCCAGCATTCCAGAC
Rap1A_R	GGGAACTTGTGCAAACCAA
Rap1B_F	CATTGTAGCTCTGAGCCAGGT
Rap1B_R	AAGGGTACCACCACAGGAAA
PRKACA_F	TGCTGGTGAAACACAAGGAG
PRKACA_R	GACGAGGAACGGAATGTTGA
PRKACB_F	TTGGGTTTGCCAAAAGAGTT
PRKACB_R	AGCCAGCTGCCATTTCATAG
**siRNA sequences**	
siNS (siNonsense)	CCAAAUUAUACCUACAUUGCU
siITGB1_1	GUGCAGAGCCUUCAAUAAA
siITGB1_2	GGUAGAAAGUCGGGACAAA
siWNT9A_1	GCAGCAAGUUCGUCAAGGA
siWNT9A_2	GCACAAGUAUGAGACGGCA
siCD44_1	GAAUAUAACCUGCCGCUUU
siCD44_2	CAAGUGGACUCAACGGAGA
siRAP1A_1	GAACAGAUUUUACGGGUUA
siRAP1A_2	GCAAGACAGUGGUGUAACU
siRAP1B_1	AAAAUACGAUCCUACGAUA
siRAP1B_2	GGACAAGGAUUUGCAUUAG
siPKACA_1	CGGAGAAUCUGCUCAUUGA

siPKACA_2	CAAGGACAACUCAAACUUA
siPKACB_1	GUUCUAUGCAGCUCAGAUA
siPKACB_2	UCAGAAUAAUGCCGGACUU
**ChIP-PCR Primer sequences**	
ACTB	TGCCCATCTACGAGGGGTAT
	ATGCCAGGGTACATGGTGGT
CD44	AGACCTCGCCCTCTCTCCAGC
	CGTCAGGACAGAGGATGACCGA
CLIC4	CAAGGGCTAGCTCAGAGCCTGAAA
	AACTCTGCCCCGGGTTCACG
ITGB1	GCCCCTGAGGAGGAGGAG
	CGGCGGCTTTAAGTGCTG
VEGFC	ACGGGCTTTCCCATGCAACTGT
	TGGATGGCGCAACATGTCCCA
WNT9A	CCCGGGAGGGGATCACGCC
	CAGCGCCCCCAACGCTCTAC
